# Hypothalamic Regulation of Brown Adipose Tissue Thermogenesis and Energy Homeostasis

**DOI:** 10.3389/fendo.2015.00136

**Published:** 2015-08-31

**Authors:** Wei Zhang, Sheng Bi

**Affiliations:** ^1^Laboratory of Psychiatry and Behavioral Sciences, Department of Psychiatry and Behavioral Sciences, Johns Hopkins University School of Medicine, Baltimore, MD, USA

**Keywords:** central nervous system, brown adipose tissue, thermogenesis, energy homeostasis, sympathetic nervous system

## Abstract

Obesity and diabetes are increasing at an alarming rate worldwide, but the strategies for the prevention and treatment of these disorders remain inadequate. Brown adipose tissue (BAT) is important for cold protection by producing heat using lipids and glucose as metabolic fuels. This thermogenic action causes increased energy expenditure and significant lipid/glucose disposal. In addition, BAT in white adipose tissue (WAT) or beige cells have been found and they also exhibit the thermogenic action similar to BAT. These data provide evidence indicating BAT/beige cells as a potential target for combating obesity and diabetes. Recent discoveries of active BAT and beige cells in adult humans have further highlighted this potential. Growing studies have also shown the importance of central nervous system in the control of BAT thermogenesis and WAT browning using animal models. This review is focused on central neural thermoregulation, particularly addressing our current understanding of the importance of hypothalamic neural signaling in the regulation of BAT/beige thermogenesis and energy homeostasis.

## Introduction

Brown adipose tissue (BAT) is a thermogenic organ that protects the body from cold environment via dissipating chemical energy (lipids and glucose) as heat. For a long period of time, BAT was thought to be only present in certain species of mammals, including rodents, hibernating animals, and newborn humans. Recently, active BAT has been found in adult humans at a cold environment (1–6). These findings have prompted investigation into the potential action of BAT in fighting against obesity and its associated metabolic disorders in humans (2, 4, 7). For instance, recent reports have shown that cold exposure (at 15–16°C for 6 h daily for 10 days or 17°C for 2 h daily for 6 weeks) can recruit human BAT and increase non-shivering thermogenesis (8) and lower body fat mass (9), although whether such effects could last for prolonged periods (months to years) is unclear. Animal studies have shown that subcutaneous transplants of embryonic BAT can reverse type I diabetes in streptozotocin-treated mice (10). The magnitude of BAT thermogenesis depends on the amount of recruitment of active BAT (11), which is influenced by various factors (3, 11, 12). Recently, several central neural modulators have been identified to reduce body weight and adiposity through promoting thermogenic activity of BAT and/or browning of white adipose tissue (WAT) to combust energy. This review will focus on the role of central nervous system (CNS), particularly hypothalamic neural signals, in the regulation of BAT thermogenesis and energy homeostasis.

### Distribution of BAT

There are two types of brown adipocytes. The classic brown adipocytes, such as the ones that reside in the interscapular region in rodents, are originated from myf5-positive cells (13). The other is the beige cells that are discretely distributed in the WAT, such as inguinal WAT in rodents (14). Beige cells are originated from the lineage of myf5-negative cells and also known as inducible or recruitable brown adipocytes in WAT (13). The amount of classic BAT in mouse strains is genetically invariant, whereas the amount of inducible brown adipocytes in WAT is more variable among strains (15). Thus, the capacity for the potential of BAT against obesogenic influence may also depend upon the genetic variability of inducible BAT in addition to classic BAT. In rodents, BAT has been found in multiple locations, including interscapular, cervical, peri-aortic, pericardiac, perirenal, and axillary regions (16, 17). In humans, BAT exists more abundantly in new born babies corresponding to the high level of non-shivering thermogenesis. The amount of BAT significantly decreases with aging as BAT mass in pediatric populations is about 10-fold higher than that of adult populations (18). Intriguingly and also most importantly, data have shown that BAT is recruitable in adult humans as cold acclimation results in great increases in BAT mass and non-shivering thermogenesis in adult humans who do not possess detectable BAT before treatment (8, 9). Human BAT is primarily distributed in the supraclavicular and neck regions, as well as in paravertebral, mediastinal, para-aortic, and suprarenal areas, but less in the interscapular area (1–5). Adult human BAT appears to contain both functional brown and beige cells. Cypess et al. have reported that although the properties of human neck fat vary substantially between individuals, human brown adipocytes share many similarities with classical rodent BAT (2). Shinoda and colleagues have further reported that adult human BAT possesses the genetic and functional features more similar to those of rodent beige cells (19).

### Function of BAT

Brown adipose tissue contains a large number of mitochondria that act as a heat generator through uncoupling protein 1 (UCP1). During BAT activation, UCP1 uncouples the oxidation of fatty acids from ATP synthesis to dissipate energy as heat. This function is very important for small mammals to maintain body temperature, especially during cold environment (11). Fatty acid oxidation (or energy combustion) in BAT could account for a great amount of total energy intake. For instance, a mouse at 5°C will have food intake approximately 3–4 times that at 30°C (20). This enormous effect on energy utilization highlights BAT as an appealing target for the prevention and treatment of obesity and other metabolic disorders.

Brown adipose tissue thermogenesis is predominately governed by the sympathetic nervous system (SNS) via the adrenergic receptor signaling pathways. Upon stimulation, sympathetic nerve releases norepinephrine that binds to β3 adrenergic receptors in the membrane of brown adipocytes to activate a cascade of signaling pathways, leading to increases in fatty acid β-oxidation and heat production (11). Recently, three subtypes of β adrenergic receptors have been found in BAT (21). Data have shown that mice lacking all three β-adrenergic receptors failed to induce BAT thermogenesis and led to rapid drop of core body temperature in response to cold exposure (22), supporting the importance of β-adrenergic receptors in thermogenesis. Particularly, during brown adipocyte development, β1-adrenergic receptors act to regulate proliferation of brown preadipocytes, while β3-adrenergic receptors mainly affect differentiation of mature brown adipocytes through a cAMP-dependent pathway (11, 23). Deficits in either of them alter BAT functions. β1-adrenergic receptor knockout mice develop hypothermia during cold exposure and exhibit impaired interscapular BAT (iBAT) thermogenesis. These mice become more susceptible to diet-induced obesity and fail to develop diet-induced thermogenesis relative to wild-type mice (21). Intriguingly, while cold induces brown adipocyte development in WAT of wild-type mice, this effect is significantly attenuated in β3-adrenergic receptor knockout mice (24), suggesting that β3 adrenergic receptors are also likely to mediate browning of WAT.

We have appreciated that BAT uses lipids as substrates for thermogenesis and activation of BAT promotes oxidative metabolism and heat production, leading to a great increase in energy utilization (11). In support of this view, recent reports have demonstrated an enormous role for BAT in triglyceride and glucose clearance. Bartelt et al. have reported that cold exposure drastically accelerated plasma clearance of triglycerides as a result of increased uptake into BAT of mice, and that in pathophysiological settings, cold exposure corrected hyperlipidemia and improved deleterious effects of insulin resistance using mouse models (25). Human studies have also shown cold-induced activation of oxidative metabolism in BAT coupled with increased uptake of non-esterified fatty acid (26). Fatty acid transport protein 1 (FATP1) likely contributes to the uptake of fatty acids in BAT as FATP1 knockout animals display smaller lipid droplets in BAT and fail to defend their core body temperature at 4°C, despite elevated levels of serum free fatty acid (27). In addition, BAT has been considered as a sink of glucose disposal. Both *in vivo* and *in vitro* studies have suggested that BAT-associated glucose uptake is regulated by norepinephrine and insulin (28–31). BAT transplantation into age- and sex-matched recipient mice increased insulin-stimulated glucose uptake (32). While glucose transporter isoform 4 (GLUT4) mediates insulin-stimulated glucose uptake in BAT in the way similar to WAT and muscle, the mechanism for norepinephrine-mediated glucose uptake is still unclear.

### Browning of WAT

Brown adipose tissue in WAT depots was initially reported by Young et al. (33), who observed that cold acclimation led to the accumulation of BAT in the parametrial fat pad. Although the intertransdifferentiation between brown and white adipocytes might be a case in fat browning (34), the identification of beige (or brite) adipocytes in WAT in both mice and humans (14, 35–37) has significantly advanced our understanding of BAT in WAT. Importantly, increased recruitment of active brown and/or beige cells in WAT has been shown to promote energy utilization/expenditure and improve glucose tolerance and insulin sensitivity. We now appreciate that browning of WAT is under the control of various key transcription factors, such as PGC-1α, C/EBPα, PPARγ, and PRDM16 (38). Growing data have also indicated the importance of CNS in fat browning (39, 40). This review will update our understanding of hypothalamic regulation of BAT thermogenesis and fat browning since the last report showing that knockdown of neuropeptide Y (NPY) in the dorsomedial hypothalamus (DMH) promotes brown adipocyte development in WAT, increases BAT activity, and elevates energy expenditure (41).

### Hypothalamic regulation of BAT thermogenesis

Although a role for the CNS in the regulation of BAT thermogenesis has long been known (11), neural circuits and chemicals underlying the central thermoregulation remain incompletely understood. Recently developed techniques for brain research have made it possible to unravel the neural mechanisms of the control of BAT thermogenesis. Using pseudorabies virus as a polysynaptic retrograde tracer, several labs have investigated potential brain areas and pathways in the modulation of sympathetic innervation of BAT and WAT. Injection of pseudorabies virus into the iBAT has revealed the sympathetic outputs from the brain to iBAT (42–44). Within the hypothalamus, viral-infected neurons were detected in the paraventricular hypothalamus (PVN), lateral hypothalamus (LH), DMH, arcuate nucleus (ARC), and preoptic area (POA), but very few or absent in the ventromedial hypothalamus (VMH) (42–45). Similarly, viral-infected neurons were found in the PVN, DMH, and POA of animals receiving pseudorabies virus injection into WAT (46). These findings suggest that the descending signals from hypothalamic areas modulate sympathetic innervation of BAT and WAT to affect BAT thermogenesis and WAT browning. The results from functional studies have provided support for this view. Using innovative approaches to manipulate specific gene and/or neuron activities in animal models, we and other investigators have identified distinct roles of hypothalamic peptides and neurons in the control of BAT thermogenesis and WAT browning. The following is primarily focused on the recent understanding of hypothalamic signaling in thermoregulation.

### POA and BAT thermogenesis

The POA is located in the rostral hypothalamus and acts as a coordinate that receives and integrates the inputs of changes in temperature from local brain and periphery system to restore thermal homeostasis in the body (47). The POA contains both warm-sensitive and cold-sensitive neurons that relay the peripheral and central thermal signals (48). The warm-sensitive neurons control both heat production and heat loss through independent pathways (49). Both shivering and non-shivering thermogenesis are affected by the signals from the warm-sensitive neurons (50, 51), which is mainly innervated by GABAergic neurons (52). Two subnucleus are identified in the POA: the median preoptic nucleus (MnPO) and the medial preoptic area (MPO) (53). For instance, inhibition of MnPO neurons completely blocks the activation of BAT thermogenesis (54), while stimulation of MPO neurons by infusion of the α-melanocyte-stimulating hormone analog melanotan II (MTII) into the MPO evokes iBAT thermogenesis (55). The POA is also activated by pyrogens, such as prostaglandin E2 (PGE2) during fever. Ablation of the PGE receptor subtype EP3 in the MnPO dramatically attenuates fever (56). Neuroanatomically, POA neuronal modulation of BAT thermogenesis and body temperature is mediated through DMH and rostral raphe pallidus (rRPa) neurons (57–59). POA neurons project to the DMH where neurons further innervate the rRPa (57, 58), and some of POA neurons also directly project to the rRPa (59). The rRPa contains sympathetic premotor neurons that serve as an important output of brain to regulate the sympathetic activity of iBAT (57–59). In support of this view, a recent report has shown that loss of portions (serotonin neurons) of this descending pathway greatly reduces BAT thermogenesis as well as WAT browning (40). Thus, the system of POA–DMH–rRPa–iBAT thermoregulation exhibits a distinct role in the control of thermal homeostasis.

### DMH and BAT thermogenesis

Earlier investigation of hypothalamic sites in thermogenesis has revealed the importance of the DMH in thermoregulation, especially by showing that the dorsal subregion of the DMH contains neurons that project to the rRPa to affect sympathetic innervation of iBAT to modulate BAT and body core temperature (60, 61). Studies have demonstrated that disinhibition of DMH neurons with the GABA receptor antagonist bicuculline methiodidide (BMI) results in increases in both core body temperature and iBAT temperature and such effects are abolished by systemic pretreatment with propranolol, a β-adrenergic receptor blocker (62). Moreover, thermogenesis evoked by activation of DMH neurons is reversed by injection of glutamate receptor antagonists in the rRPa (63). These data indicate that DMH neurons are tonically inhibited by GABAergic inputs and disinhibition of DMH neurons (resulting in activation of glutamatergic neurons) promotes glutamatergic descending signals to the rRPa to elevate subsequent sympathetic activity to iBAT. Recent studies have further demonstrated a specific role for leptin in thermoregulation through acting on dorsal DMH neurons in mice. Activation of LepRb neurons in the DMH/DHA promotes BAT thermogenesis, and intra-DMH/DHA injections of leptin normalize hypothermia and reduce body weight gain in *ob/ob* mice (64). Furthermore, prolactin-releasing peptide (PrRP) neurons in the DMH have been shown to mediate the thermogenic effect of leptin. Disruption of LepRb selectively in PrRP neurons blocks thermogenic responses to leptin and causes obesity in mice (65). Data have also shown that leptin can directly act on NPY-expressing neurons in the DMH (specifically in the non-compact subregion) in diet-induced obese mice, but it is puzzling that these NPY neurons do not contain LepRb (66). In contrast to mice, lepRb-expressing neurons are found in the ventral subregion of the DMH, but undetectable in the dorsal area in rats (67), implying that the nature of dorsal DMH neurons in modulating BAT thermogenesis likely differs between mice and rats or other species. We have recently found that NPY-expressing neurons in the compact subregion of the DMH play a significant role in energy homeostasis in rats via affecting both food intake and energy expenditure. Overexpression of NPY in the DMH increases food intake and body weight, leading to obesity and type 2 diabetes in the Otsuka Long Evans Tokushima fatty (OLETF) rats. These phenotypes can be rescued by NPY knockdown in the DMH via adeno-associated virus (AAV)-mediated NPY-specific RNAi (68). Furthermore, DMH NPY knockdown increases iBAT thermogenesis and results in browning of WAT in subcutaneous inguinal fat in Sprague-Dawley rats and prevents diet-induced obesity (41). A neuroanatomical analysis of DMH NPY and LepRb-expressing neurons has revealed that DMH NPY neurons do not contain lepRb and that *Npy* expression in the DMH is not affected by alterations in circulating leptin levels in rats (67), indicating that DMH NPY is not under the control of leptin in rats. Given that the dorsal DMH contains NPY Y1 and Y5 receptors, we speculate that DMH NPY may modulate neuronal signaling in the dorsal DMH neurons to affect BAT thermogenesis.

### VMH and BAT thermogenesis

Several lines of evidence have suggested the involvement of the VMH in BAT thermogenesis. Lesion studies have shown that the rats with VMH lesions fail to respond to both cold and prostaglandin E1 (PGE1)-induced thermogenesis in iBAT (69, 70). However, data from the studies of the effects of electrical or chemical stimulation of the VMH on BAT thermogenesis provide controversial results. Some groups have shown that electrical stimulation of the VMH causes an increase in iBAT thermogenesis (71) and elevates blood flow to the BAT, which is an important contributor to its thermogenesis (72). However, Halvorson and colleagues have found that both electrical and chemical stimulation of the VMH increase iBAT thermogenesis only in rats acclimated to 4°C but not 21°C (73). DiMicco’s group has further reported that chemical stimulation of the DMH, but not the PVN and VMH, evokes non-shivering thermogenesis in rats (62).

Despite these discrepancies, recent studies at molecular levels provide support for the role of the VMH in thermoregulation. One report has shown that estradiol inhibits AMP-activated protein kinase (AMPK) through the estrogen receptor α (ERα) in the VMH and results in activation of BAT thermogenesis through the SNS (74). Studies have also shown that stimulation of the glucagon-like peptide-1 (GLP-1) system with GLP-1 analogs in the VMH increases BAT thermogenesis and WAT browning, leading to decreased body weight in a feeding-independent manner (75). Such effects can be normalized by activation of AMPK signaling using viral-mediated constitutive active AMPKα (75). Bone morphogenetic proteins (BMPs) have been found to regulate both differentiation of BAT progenitor cells and physiological function of mature brown adipocytes. BMP8 upregulates expression of UCP1 and other genes associated with mitochondriogenesis and β-oxidation of fatty acids through BMP8b receptors. BMP8b receptors are highly expressed in the VMH (76). BMP8B activate BAT thermogenesis through increasing BAT response to adrenergic stimulation as well as brain sympathetic outflow to BAT via deactivation of hypothalamic AMPKα likely mediated by VMH BMP8b receptors (76). Together, these findings suggest that the VMH also contributes to the regulation of BAT thermogenesis. Since viral-infected neurons are scarcely detected in the VMH of animals receiving pseudorabies virus injection into iBAT as descried above, one possibility for the action of VMH neurons in modulating BAT thermogenesis is through a neuroendocrine reflex system.

### ARC and BAT thermogenesis

The ARC has been well identified as one of the most important hypothalamic nuclei that affect energy homeostasis. It contains both anorexigenic neurons (POMC and CART) and orexigenic neurons (NPY and AGRP). Food restriction causes significantly increased expression of NPY and AgRP in the ARC, while food restriction also decreases adaptive thermogenesis (77), implying ARC NPY and AgRP in thermoregulation. In support of this view, recent studies have found that ARC NPY suppresses sympathetic outflow to iBAT and lowers BAT thermogenesis via Y1 receptor-mediated reduction of tyrosine hydroxylase expression in the PVN (78). These effects are independent of changes in body weight and physical activity (78). Recent evidence has also demonstrated the importance of AgRP neurons in thermoregulation and specifically, data have shown that inactivation of AgRP neurons promotes retroperitoneal WAT browning and protects mice against diet-induced obesity and insulin resistance (79). This effect is mediated by a key enzyme called *O*-linked β-*N*-acetylglucosamine (*O*-GLcNAc) transferase (79). *O*-GLcNAc levels in AgRP neurons are elevated in response to fasting, at a condition when thermogenesis is suppressed, whereas genetic ablation of *O*-GlcNAc transferase in AgRP neurons limits fasting-induced suppression of thermogenesis, suggesting that *O*-GlcNAc signaling in AgRP neurons is essential for suppressing thermogenesis to conserve energy in response to fasting (79). Since NPY and AgRP are co-localized in ARC neurons and data have shown that the PVN mediates the thermogenic effect of ARC NPY (78), ARC AgRP regulation of BAT thermogenesis is also likely to be mediated through the PVN. On the other hand, POMC neurons in the ARC receive the adiposity signals leptin and insulin corresponding to energy status and exhibit an anorectic effect on energy balance. Dodd et al. recently report that a combined action of leptin and insulin on POMC neurons promotes WAT browning and energy expenditure and prevents diet-induced obesity through protein tyrosine phosphatase 1B (PTP1B) and T cell protein tyrosine phosphatase (TCPTP) signaling as deletion of the phosphatases PTP1B and TCPTP enhanced insulin and leptin signaling in POMC neurons (80). Given that ARC POMC and NPY/AgRP neurons are functionally antagonistic, the next logic step would be of interest to determine whether the ARC–PVN neural pathway contributes to this POMC neuron-mediated thermogenic action.

### LH and BAT thermogenesis

The neurochemical characterization of the hypothalamic projections to the BAT in rats identifies the presence of melanin-concentrating hormone (MCH) and orexin (hypocretins) neurons in the caudal aspect of LH (43), implicating MCH and orexin neurons in thermoregulation. In support of this view, MCH knockout mice have elevated levels of UCP1 protein by threefold along with an increase in energy expenditure as compared to control mice (81). The next question would be important to evaluate whether MCH-containing neurons in the LH contribute to this effect and its underlying neural circuits. Orexin neurons in the LH play a critical role in both arousal and energy balance. A recent study has demonstrated that orexin-null mice displayed impaired BAT differentiation and function, which can be normalized by the injection of orexin to the orexin-null dams (82). Moreover, data have shown that orexin neurons in the perifornical lateral hypothalamus (PeF/LH) are positively correlated with sympathetic outflow to iBAT and BAT thermogenesis (83), although such effects appear unexpected as the orexigenic peptide orexin would produce a negative action on BAT thermogenesis and energy expenditure. Nevertheless, recent evidence indicates the importance of LH signaling in overall thermoregulation.

### PVN and BAT thermogenesis

The PVN plays a critical role in the modulation of energy balance. As discussed above, PVN neurons serve as an important mediator of ARC neural regulation of energy expenditure. Shi and colleagues have found that tyrosine hydroxylase expressing neurons in the PVN mediate ARC NPY-induced decrease in BAT thermogenesis (78). Data from pharmacological studies have also provided support for a role of PVN melanocortin in thermogenesis. Intra-PVN injection of melanocortin receptor agonist MTII significantly increased energy expenditure (oxygen consumption) in mice (84). Song et al. have shown that melanocortin-induced activation of PVN neurons affects sympathetic outflow to iBAT and BAT thermogenesis (85). Acute parenchymal microinjection of MTII into the PVN increases iBAT temperature in hamsters (85). These results indicate that PVN melanocortin affects BAT thermogenesis. By contrast, genetic studies provide mixed results. Using PVN-selected single-minded 1 (Sim1)-Cre transgenic mice, Balthasar et al. have reported that genetic restoration of MC4R expression in the PVN of *Mc4r* null mice reversed hyperphagia, but did not affect energy expenditure (86), whereas Xu and colleagues have found that MC4Rs on Sim1 neurons in the PVN regulate both energy expenditure and food intake (87). Particularly, they have shown that while the restoration of MC4Rs in Sim1 neurons in the PVN dramatically reduced obesity of Mc4r-null mice, selective disruption of glutamate release from these MC4R neurons prevented this reversal effect by affecting both energy expenditure and food intake, further indicating that glutamate mediates the function of MC4Rs on PVN Sim1 neurons in thermoregulation as well as feeding control (87). In addition, previous work has suggested that ARC POMC and NPY/AGRP neurons project to GABA interneurons in the PVN to coordinately regulate GABA release and thereby affect food intake and energy expenditure (84). Xu and colleagues have found that MC4Rs are largely co-localized with vesicular glutamate transporter 2 (VGLUT2), but few with vesicular GABA transporter (VGAT) in PVN Sim1 neurons, suggesting that most MC4R neurons in these PVN regions are glutamatergic (87). Nevertheless, one explanation is that the PVN contains both populations of neurons (glutamatergic and GABAergic) that integrate the inputs of melanocorin agonist and antagonist directly or indirectly to control food intake and energy expenditure.

## Hypothalamic Thermoregulation in Coping with Environmental Challenges

### Cold-induced BAT thermogenesis

Physiologically, when the ambient temperature falls below the thermoneutral zone (cold environment), BAT is activated to produce heat to maintain body temperature in mammals. As discussed above, studies on animal models have shown that the hypothalamus serves as an essential site in this thermoregulation. Particularly, the POA/DMH/rRPa neural system is excited to promote the sympathetic activity in BAT to increase BAT and body temperature in response to cold exposure (47, 61). Using c-Fos as a marker of neuronal activation, other hypothalamic areas, such as the PVN, ARC, and LH, and extra-hypothalamic regions, such as the nucleus of the solitary tract (NTS), have also been identified in the rodent brain in response to cold (59), indicating that cold exposure causes neuronal activation at multiple brain sites, suggesting that these areas may also contribute to thermoregulation due to cold environment. Data have shown that animals with cold exposure become hyperphagic (20), indicating that animals increase their energy intake to meet energy demands derived from increased energy expenditure, i.e., cold exposure actually elevates both thermal sensory (directly) and feeding regulatory (secondarily) neuronal activities. For instance, hypothalamic orexigenic peptides (such as ARC AgRP/NPY) would be elevated while anorectic peptides (such as POMC) would be decreased to promote energy intake to restore energy balance. Thus, although ARC AgRP/NPY and POMC neurons play an important role in the regulation of energy expenditure, these neural systems do not appear to contribute to cold-induced thermogenesis physiologically. Similarly, we have found that knockdown of NPY in the DMH enhanced cold-induced thermogenesis via increased BAT thermogenesis, but NPY gene expression is actually increased in the DMH of intact rats in response to cold exposure. Thus, these data support the view that physiologically, the hypothalamic systems are organized in the way to integrate central and peripheral signals (derived from changes in energy status) to maintain energy homeostasis. Recently, cold exposure has been shown to promote recruitment of active brown and/or beige adipocytes in fat depots of adult humans (1–6). Lean subjects have increased BAT thermogenesis much greater than obesity subjects by exploring to a cold environment, suggesting that loss of active BAT in obese individuals may become a contribute factor to obesity pathologically. Do the hypothalamic neural systems in the overall control of energy balance also go awry in these individuals? In other words, while thinking of cold-induced recruitment of active BAT for fighting against obesity, adding additional activation of SNS outflow to BAT through manipulating hypothalamic neural activities, such as knockdown of DMH NPY, could provide an important route for the effective control of body weight.

### Diet-induced BAT thermogenesis

Diet-induced thermogenesis in BAT was initially reported in 1979 by Rothwell and Stock who showed that rats fed high energy cafeteria diet (containing high fat and sugar) had increased body core and BAT temperature (88). This effect appears diet selective as the thermogenic capacity of BAT was reduced in rats fed a high protein, carbohydrate-free diet (89). Similar to the SNS mediation of cold-induced BAT thermogenesis, β-adrenergic receptors are necessary for diet-induced thermogenesis because mice lacking β-adrenergic receptors developed massive obesity that was due entirely to a failure of diet-induced thermogenesis (22). In contrast to well-studied cold-induced thermogenesis (61), the central neuromodulation of diet-induced thermogenesis remains less understood. Feeding studies have shown that signals arisen from peripheral metabolic changes can be relayed to the CNS through vagal afferent signaling pathways during the control of food intake. Recent studies have demonstrated the importance of this vagal signaling in BAT thermogenesis. Blouet and Schwartz reported that duodenal lipid sensing activates vagal afferents to regulate BAT thermogenesis in rats (90). This effect can be blocked by systemic administration of the cholecystokinin (CCK)-1 receptor antagonist or parenchymal administration of the glutamate *N*-methyl-d-aspartate receptor blocker MK-801 directly into the caudomedial nucleus of the solitary tract (NTS), indicating that the CCK–NTS signaling also mediates diet-induced BAT thermogenesis. Intriguingly, peripheral administration of CCK activates neurons in the NTS, as well as within the PVN and the DMH using c-Fos as an activation marker (91), suggesting that neurons in the PVN and the DMH may modulate diet-induced thermogenesis as well as control food intake.

### Stress-induced BAT thermogenesis

Stress has long been recognized to cause thermogenesis or hyperthermia. Forced immobilization stress results in increased heat production in BAT and this effect is prevented by sympathetic denervation of BAT (92), indicating that stress-induced BAT thermogenesis is mediated through the SNS. Further studies have shown a cross adaptive thermogenesis between cold and stress. Repetitive immobilization stress improves cold tolerance. This improvement is likely through enhancing the capacity of BAT thermogenesis because noradrenaline (NA) turnover of BAT was greatly increased by both stress and cold challenges (93). Despite these observations, the central neural mechanism underlying the effect of stress on BAT thermogenesis has yet to be explored till recently. Given that the DMH is an important site for autonomic responses to stress stimuli (94), Kataoka and colleagues have demonstrated that the DMH–rRPa neural pathway also mediates a psychosocial stress-induced thermogenesis in BAT (95). Inactivation of DMH neurons via muscimol prevents stress-induced increases in BAT and body temperature (95). Within the rRPa, both glutamatergic and serotonergic neurons are involved in psychosocial stress-induced BAT thermogenesis and hyperthermia as injection of glutamate receptor antagonists or a 5-HT_1A_ receptor agonist eliminated these thermogenic effects induced by social defeat stress (95). Moreover, in response to repetitive insertion of a temperature probe into their rectum (handling stress), prepro-orexin knockout mice showed a normal temperature change as compared to that of wild-type littermates (WT), while orexin neuron-ablated mice showed an attenuated response, suggesting that neurotransmitters other than orexin in orexin neurons play an important role in stress-induced non-shivering thermogenesis (96). Furthermore, data have shown that in addition to its feeding effect, NPY plays a pivotal role in modulating various stress responses (97), but a particular role for hypothalamic NPY in the regulation of stress-induced hyperthermia is undetermined. We have recently found that knockdown of NPY in the DMH promotes BAT thermogenesis, elevates energy expenditure, and enhances cold stress response (98). Thus, DMH NPY signaling might also contribute to stress-induced thermogenesis.

## Summary and Perspective

**Figure 1:**
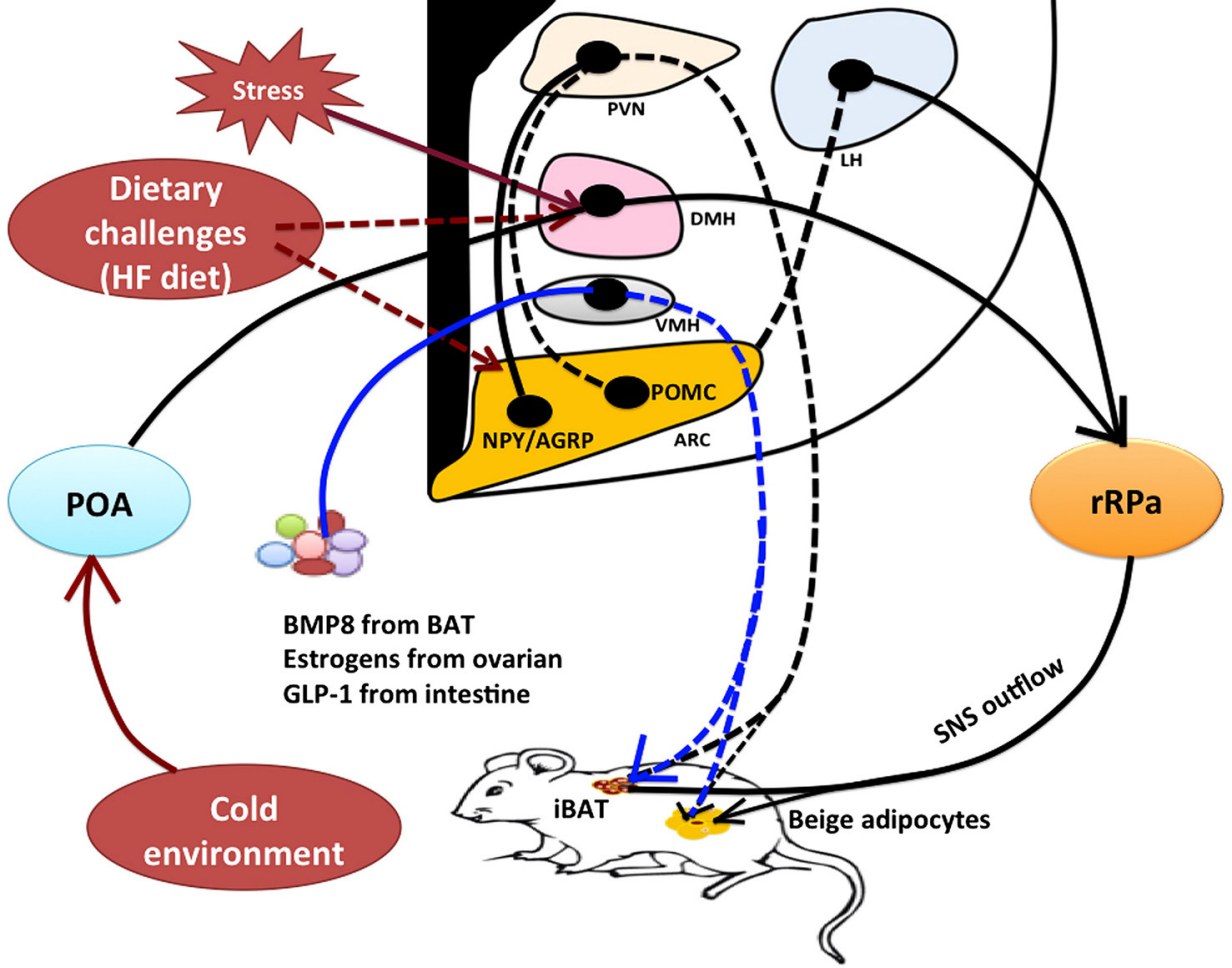
**Model of hypothalamic neural regulation of BAT thermogenesis**. Within the hypothalamus, the POA contains thermal-sensitive neurons that receive and integrate thermal sensory signals from cold exposure to promote BAT thermogenesis through the POA–DMH–rRPa pathway. The rRPa contains sympathetic premotor neurons that relay central thermal signals from the POA and DMH to affect sympathetic activity of BAT to produce heat. The DMH and rRPa pathway also contributes to the central regulation of stress-induced BAT thermogenesis and hyperthermia. Stress stimulation activates thermoregulatory neurons within the DMH thereby affecting sympathetic input to the rRPa to cause thermogenesis, but the upstream neural pathway from the DMH remains unclear. The hypothalamus contains both orexigenic and anorexigenic neurons, such as NPY neurons in the DMH, NPY/AGRP neurons and POMC neurons in the ARC, and orexin and melanin-concentrating hormone (MCH) neurons in the LH. These neurons are also involved in thermoregulation. Excessive high-fat diet likely results in alterations to these neuronal activities to increase energy expenditure (BAT thermogenesis) in order to restore energy balance, but neural circuits contributing to diet-induced thermogenesis have yet to be well characterized. Furthermore, the hypothalamus can mediate the effects of peripheral signals on BAT thermogenesis likely through a neuroendocrine fashion. For instance, BMP8 from BAT, estrogens from ovaries, and GLP-1 from intestine all have a significant impact on BAT thermogenic action through the VMH. Both classical interscapular brown adipose tissue (iBAT) and beige/brite cells in the white adipose tissue (WAT) are under the control of sympathetic nervous system (SNS). *Solid lines or arrows indicate the known pathways, and dash lines or arrows indicate unknown pathways. BAT, brown adipose tissue; iBAT, interscapular brown adipose tissue; POA, preoptic area; DMH, dorsomedial hypothalamus; rRPa, rostral raphe pallidus; NPY, neuropeptide Y; AGRP, agouti-related peptide; POMC, proopiomelanocortin; BMP8, bone morphogenetic protein 8; GLP-1, glucagon-like peptide-1; PVN, paraventricular hypothalamus; LH, lateral hypothalamus; VMH, ventromedial hypothalamus; ARC, arcuate nucleus; HF diet, high-fat diet.

The findings of active BAT (brown and beige cells) in adult humans have provided the potential for BAT in combating obesity and associated comorbidities. Using animal models, we now appreciate that the brain regulates sympathetic outflows to BAT to modulate BAT thermogenesis and body temperature. Especially, recent studies have revealed that hypothalamic peptide signaling plays an important role in the control of BAT thermogenesis and WAT browning in rodents (Figure 1). Physiologically, the hypothalamic neural system integrates central and peripheral signals of energy status to regulate both food intake and energy expenditure to maintain energy homeostasis. Since obese individuals have lost or have very low levels of active BAT, the critical question in the aspect of the relationship between inactive BAT and hypothalamic thermoregulation under the obesity condition needs to be addressed. For example, how does impaired hypothalamic signaling cause ineffective recruitment of active BAT in obese individuals? Can pathological changes in metabolic syndrome result in dysfunction of hypothalamic control of BAT recruitment? Dysfunctional BAT thermogenesis leads to loss of the capacity of BAT to regulate body weight. Thus, the complete characterization of the physiological and pathological roles of the hypothalamus in the overall control of food intake and energy expenditure will significantly advance our understanding of the hypothalamic-mediated BAT thermoregulation system, promoting the strategies for the better development of pharmaceutical drugs for the treatment of obesity and metabolic disorders.

## Conflict of Interest Statement

The authors declare that the research was conducted in the absence of any commercial or financial relationships that could be construed as a potential conflict of interest.

## Abbreviations

ARC, arcuate nucleus; BAT, brown adipose tissue; CNS, central nervous system; DMH, dorsomedial hypothalamus; LH, lateral hypothalamus; POA, preoptic area; PVN, paraventricular hypothalamus; rRPa, rostral raphe pallidus; SNS, sympathetic nervous system; VMH, ventromedial hypothalamus; WAT, white adipose tissue.
